# Supplementary material for: Effect of Climate on Bacterial and Archaeal Diversity of Moroccan Marine Microbiota
Source: Microorganisms. 2022 Aug 10;10(8):1622. doi: 10.3390/microorganisms10081622 (PMC9414901; doi:10.3390/microorganisms10081622)
Supplement: Supplementary file 1 [file microorganisms-10-01622-s001.zip › microorganisms-1834389-supplementary.pdf]

SAMPLE HANDLING SHEET FOR A SEA WATER SAMPLE

Date: ——— / ——— / ———

Sample Identification

Time of sampling:

Laboratory time arrival:

ID:

Water temperature:

Environnment temperature:

Sample origin

Nature of sampled water:

Sampling point:

State:

Region:

Localization in relation to a well, spring, river, water treatment plant...:

Sampling conditions:

Physical apparence :

Color:
Aspect:
Smell:
Turbidity:
Remarks:

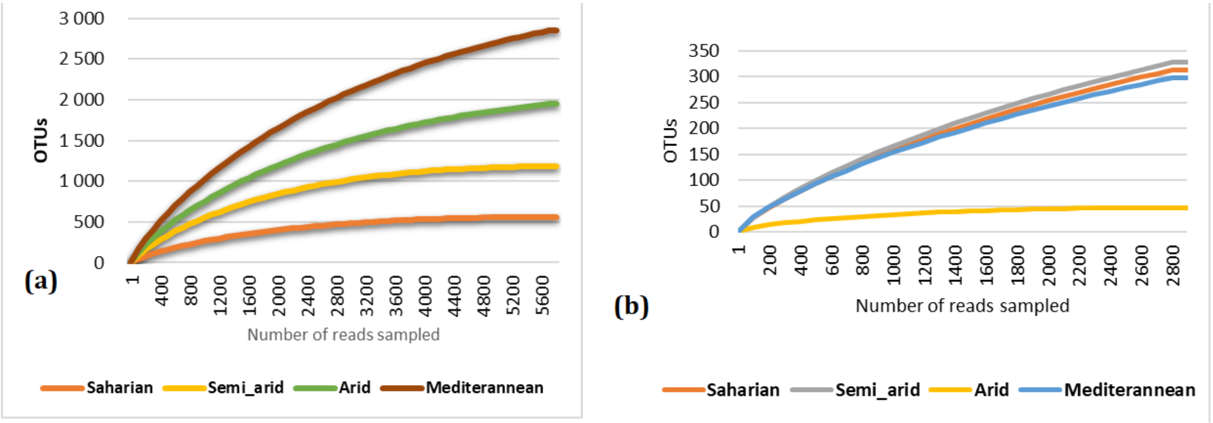

Figure S1: Rarefaction curves of bacterial OTUs (a) and archaeal OTUs (b) of the samples based on the 16SrRNA gene amplicon sequencing**.**

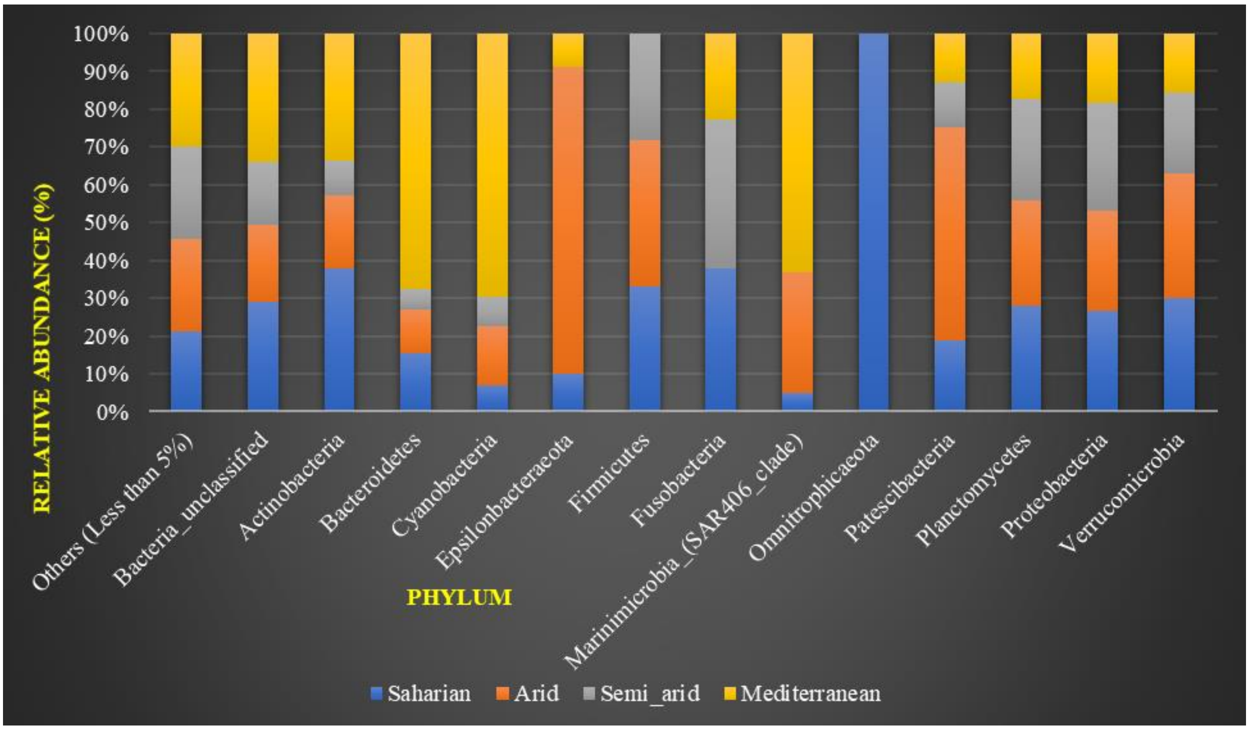

Figure S2: Distribution of the most abundant bacterial phyla based on 16S rRNA gene amplicon sequencing in the microlayer of marine waters. Phyla contributing to less than 5% to each sample are not involved.

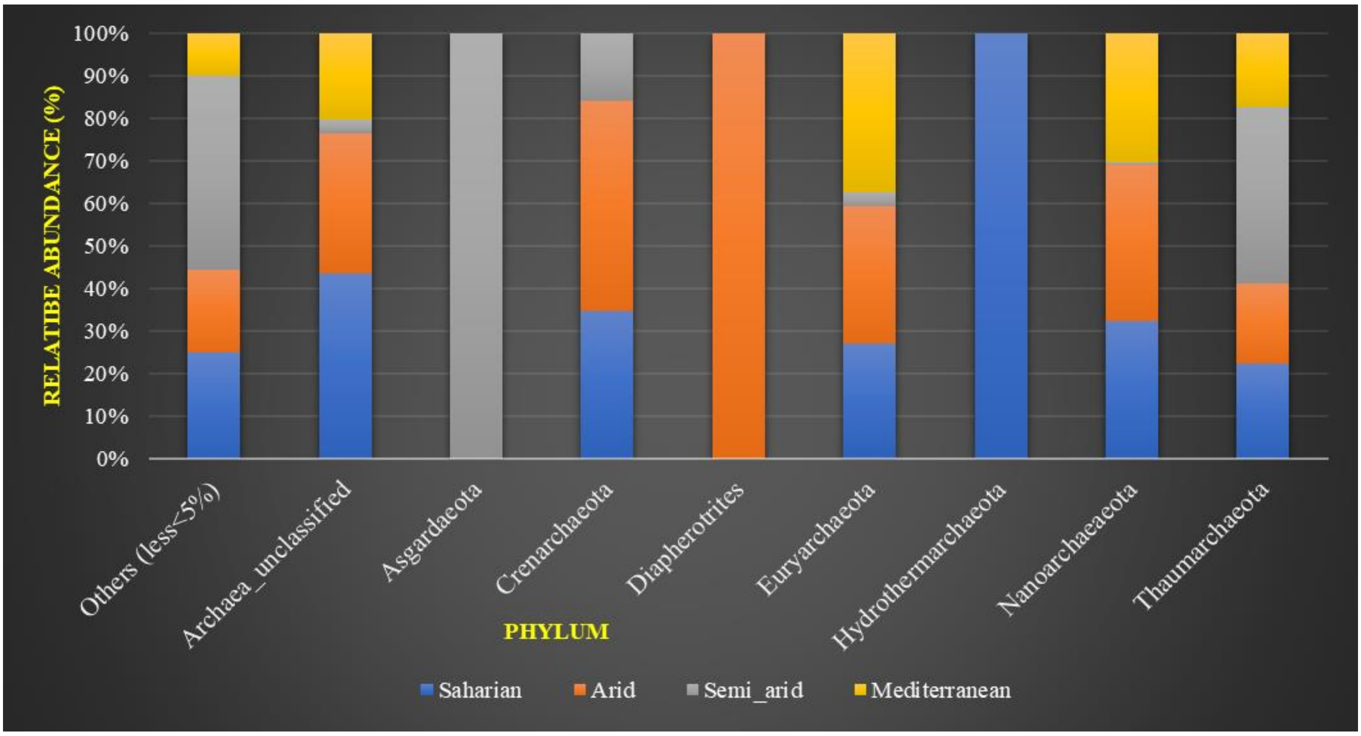

Figure.S3: Distribution of the most abundant archaeal phyla based on 16S rRNA gene amplicon sequencing in the surface microlayer of marine waters.
